# Zidovudine (AZT) Monotherapy Selects for the A360V Mutation in the Connection Domain of HIV-1 Reverse Transcriptase

**DOI:** 10.1371/journal.pone.0031558

**Published:** 2012-02-21

**Authors:** Jessica H. Brehm, Yanille Scott, Dianna L. Koontz, Steven Perry, Scott Hammer, David Katzenstein, John W. Mellors, Nicolas Sluis-Cremer, for the AIDS Clinical Trials Group Study 175 Protocol Team

**Affiliations:** 1 Division of Infectious Diseases, Department of Medicine, University of Pittsburgh School of Medicine, Pittsburgh, Pennsylvania, United States of America; 2 Department of Infectious Disease and Microbiology, University of Pittsburgh Graduate School of Public Health, Pittsburgh, Pennsylvania, United States of America; 3 Columbia University Medical Center, New York, New York, United States of America; 4 Division of Infectious Diseases, Center for AIDS Research, Stanford, California, United States of America; Burnet Institute, Australia

## Abstract

**Background:**

We previously demonstrated *in vitro* that zidovudine (AZT) selects for A371V in the connection domain and Q509L in ribonuclease H (RNase H) domain of HIV-1 reverse transcriptase (RT) which, together with the thymidine analog mutations D67N, K70R and T215F, confer greater than 100-fold AZT resistance. The goal of the current study was to determine whether AZT monotherapy in HIV-1 infected patients also selects the A371V, Q509L or other mutations in the C-terminal domains of HIV-1 RT.

**Methodology/Principal Findings:**

Full-length RT sequences in plasma obtained pre- and post-therapy were compared in 23 participants who received AZT monotherapy from the AIDS Clinical Trials Group study 175. Five of the 23 participants reached a primary study endpoint. Mutations significantly associated with AZT monotherapy included K70R (p = 0.003) and T215Y (p = 0.013) in the polymerase domain of HIV-1 RT, and A360V (p = 0.041) in the connection domain of HIV-1 RT. HIV-1 drug susceptibility assays demonstrated that A360V, either alone or in combination with thymidine analog mutations, decreased AZT susceptibility in recombinant viruses containing participant-derived full-length RT sequences or site-directed mutant RT. Biochemical studies revealed that A360V enhances the AZT-monophosphate excision activity of purified RT by significantly decreasing the frequency of secondary RNase H cleavage events that reduce the RNA/DNA duplex length and promote template/primer dissociation.

**Conclusions:**

The A360V mutation in the connection domain of RT was selected in HIV-infected individuals that received AZT monotherapy and contributed to AZT resistance.

## Introduction

Zidovudine (3′-azido-3′-deoxythymidine, AZT) was the first antiviral drug approved by the U.S. Food and Drug Administration for the treatment of HIV infection. AZT is a nucleoside reverse transcriptase (RT) inhibitor (NRTI) that, after metabolism by cellular kinases to its triphosphate form (TP) in cells, competes with the natural substrate TTP for binding and incorporation by HIV-1 RT into the nascent viral DNA. Because AZT lacks a 3′-OH on the ribose sugar, its incorporation into viral DNA results in chain-termination.

HIV-1 resistance to AZT was first reported in 1989 [1]. Resistance was conferred by mutations in the polymerase domain of RT that included D67N, K70R, T215Y/F and K219Q. Subsequently, two additional AZT resistance mutations were identified: M41L and L210W [2], [3], [4]. After the discovery that d4T (2′,3′-didehydro-2′,3′-dideoxythymidine) could select the same mutations, the term thymidine analog mutations (TAM) was adopted to reflect their role in resistance to both AZT and d4T. In general, each TAM alone confers between 1.5- to 4-fold resistance, and multiple mutations are required for high-level resistance [5]. The most common combination of mutations selected includes M41L, L210W and T215Y and excludes K70R (TAM-1 pathway). A second pattern includes D67N, K70R, T215F and K219Q/E (TAM-2 pathway) [5]. Biochemical studies have demonstrated that RT containing TAMs shows an increased capacity to unblock AZT-monophosphate (MP) terminated primers in the presence of physiological concentrations of ATP [6], [7]. In this regard, structural studies have shown that TAMs enhance the binding and/or placement of ATP in the HIV-1 RT active site [8].

Recent studies have demonstrated that AZT resistance can be increased by mutations in the connection domain of HIV-1 RT. For example, the G333D/E, G335C, N348I, A360V/I, T369V and A371V mutations have all be shown to augment AZT resistance alone or in combination with TAMs [9], [10], [11], [12]. However, all of these mutations were identified in RT sequences from participants exposed to multiple RT inhibitors and it is not known if these connection domain mutations were selected specifically by AZT. For example, Yap *et al* reported that the N348I mutation is highly associated with TAMs, the lamivudine mutation M184V/I, and the non-nucleoside inhibitor resistance mutations K103N and Y181C/I [13]. Consistent with this observation, mechanistic analyses suggest that this mutation may compensate for the antagonism of TAMs by M184V/I and Y181C [14], [15].

We previously carried out *in vitro* selection experiments by serial passage of HIV-1 in increasing concentrations of AZT to determine whether AZT selects mutations in the connection and/or ribonuclease H (RNase H) domains of RT [16]. Two novel mutations – A371V in the connection domain and Q509L in the RNase H domain – were selected in combination with D67N, K70R and T215F that together conferred greater than 100-fold AZT resistance. The goal of the current study was to determine whether AZT monotherapy in HIV-1 infected participants selects the A371V, Q509L or other mutations in the connection or RNase H domains of RT.

## Results

### Changes in serum HIV-1 RNA with AZT monotherapy

Pre-therapy and last-on-therapy serum samples were available from 5 participants who reached a primary study endpoint (see Methods) on AZT monotherapy and from 18 participants who did not reach a primary study endpoint on AZT monotherapy. The mean pre-therapy HIV-1 RNA concentration of participants reaching a study endpoint was 33,261 copies/mL (range 3,650–67,134 copies/mL), which was not significantly different from that of participants not reaching a study endpoint (mean 41,002 copies/mL; range 1,439–168,576 copies/mL; p = 0.875). There was a strong trend (p = 0.06) toward higher serum HIV-1 RNA concentration in last-on-therapy samples from participants reaching a primary study endpoint (mean 87,264 copies/mL; range 35,227–216,219 copies/mL) compared with those who did not (mean 37,424 copies/mL; range 826–138,776 copies/mL).

### Mutations selected by AZT monotherapy

Table 1 shows the mutations in HIV-1 RT that emerged in participants who received AZT monotherapy. TAMs in the polymerase domain of RT emerged in 5 of 5 participants reaching a study endpoint compared with 11 of 18 (61%) of participants who did not reach a study endpoint. Although each of the known TAMs was identified in one or more on-treatment samples, only the K70R and T215Y mutations were significantly associated with AZT monotherapy. Of note, the A360V in the connection domain of HIV-1 RT was also significantly associated with AZT monotherapy and was more frequent than the M41L, D67N, L210W or K219E/Q mutations. A360V emerged in 2 of 5 participants who reached a study endpoint and in 4 of 18 participants who did not. Among the 6 on-treatment samples that contained A360V, it was a pure mutant (100%) in 2 samples and a mixture (<50%) in 4 samples, as assessed by population sequencing (Table 2). Analysis of longitudinal samples in the 4 participants who did not reach study endpoint revealed that A360V generally emerged at the same time as TAMs, although in one participant it was the first mutation to emerge (Table 3). In one of the participants who reached a study endpoint, A360V emerged at the same time as TAMs, however in the other participant it clearly emerged after TAMs (Table 3). The connection domain mutation A371V was more frequent in last-on-therapy samples compared to pre-therapy samples, but this difference was not statistically significant (p = 0.25). The N348I and Q509L mutations were not detected in any of the 23 on-treatment samples.

**Table 1:** Mutations selected by AZT monotherapy.

Domain	Mutation	% Pre Therapy (N)	% AZT Monotherapy (N)	p-value^a^
Polymerase^b^	M41L	0.0% (0)	13% (3)	0.250
	D67N	0.0% (0)	22% (5)	0.063
	K70R	4.3% (1)	52% (12)	<0.001
	L210W	0.0% (0)	4.3% (1)	1.0
	T215Y	0.0% (0)	30% (7)	0.016
	K219E/Q	0.0% (0)	13% (3)	0.250
Connection	A360V	0.0% (0)	26% (6)	0.031
	A371V	4.3% (1)	17% (4)	0.250

a Two-sided McNemar's exact test between pre-therapy and AZT-experienced (N = 23 pairs). Not corrected for multiple comparisons.

b TAMs listed in the IAS-USA 2010 drug resistance tables.

**Table 2:** Mutations and polymorphisms associated with A360V.

Sample ID	HIV-1 RT Domain		
	Polymerase	Connection	RNase H
3^a^	V35L, T39A, **M41L** b, E42E/K, R83K, E122K, **L210L/W**, L214F, **T215Y**, K275R, R277K, E291D, E297K	E328D, I329V, **A360V**, T376A, T377S, V435I	S447N, T468S, L491S, K540K/R, N519S
4	V35I/V, **D67N**, **K70R**, R83K, K104K/N, Q207E, R211K, L214F, **T215I**, **K219E**, V245E, P272A, R277K, T286A, E297K	I329L, T338S, R356K, T357M, **A360A/V**, E399D	A446S, S447N, T468S, L491S, N519C/S/T, A554T
4 clone^c^	V35I, **D67N**, **K70R**, R83K, Q207E, R211K, L214F, **T215I**, **K219E**, V245E, P272A, R277K, T286A, E297K	I329L, T338S, R356K, T357M, **A360V**, V365I, E399D	A446S, S447N, D460N, T468S, L491S, N519T, A554T
6	**K70R**, R83K, E122K, D123D/E, S162C/Y, I178I/L, R211K, L214F, R277K, K281R, T286A/T, E297K	I329L, F346F/H/L/Y, R356K/R, T357M, **A360A/V**, T376S, T377S/T, V381I, E399D/E, T400I/T	V435I, S447N, T468S, N519S, A554T
11	K20K/R, **D67D/N**, T69S, **K70R**, K103K/R, Q174K, Q207E, R211K, **K219K/Q**, V245M, P272A, R277K, I293V, E312A	G333E, Q334H, **A360V**, K390R, Q394P/Q, V435I/V	S447N, K451K/R, L452L/V, N460D, T468P, K512R, N519S, A554A/S, V559I/V
11 clone	T69S, **K70R**, Q174K, Q207E, R211K, V245M, K263R, P272A, R277K, I293V, E312A	G333E, Q334H, **A360V**, K390R, V435I	S447N, K451R, N460D, T468P, K512R, N519S
29	T69S, **K70R**, R83K, E122K, S162C/Y, L214F, P272A, Q278H, T286A, K311K/R	T357M, G359S, **A360A/V**, T376A	S447N, T468S, L491S, N519S
31	**K70K/R**, E122K, D123E, D177E, T200A/T, Q207E, R211K, L214F, V245K, T253A/T, L282L/P, V292I, I293V	Q334L, T357M, **A360A/V**, T376A, T400A, E404D	S447N, L452E/L/V, N460D, T468P, N519N/S, K527N, K530K/R, A554A/T, V559I/V

a Mutations/polymorphisms of the viral population in each participant sample compared to consensus subtype B RT (Los Alamos HIV Sequence Database);

b TAMs highlighted in bold;

c Mutations/polymorphisms of an individual participant-derived recombinant clone when compared to consensus subtype B RT (Los Alamos HIV Sequence Database).

**Table 3:** Pattern of emergence of resistance mutations among participants who selected A360V.

Participant #	Reached Study Endpoint?	Pre-Therapy (Week 0)	Earliest Sample (Week)^a^	Last -On-Therapy Sample (Week)^b^
3	Yes	None	K70K/R, T215S/T, **A360A/V** c (20)	M41L, T215Y, **A360V** (104)
4	Yes	None	D67N, K70R, T215I/T, K219E/K (32)	D67N, K70R, T215I, K219E, **A360A/V** (56)
6	No	None	**A360A/V** c (20)	K70R, **A360A/V** (44)
11	No	None	K70K/R, T215S/T, **A360A/V** c (20)	D67D/N, K70R, K219K/Q, **A360V** (152)
29	No	None	K70R, **A360A/V** (32)	K70R, **A360A/V** (128)
31	No	None	None (8)	K70R, **A360A/V** (20)

a Sample obtained at earliest available time point after treatment initiation and which mutations were present compared with the pre-therapy sample.

b Sample obtained at time point of last-on-therapy sample available. Participants 3 and 4 reached a study endpoint. Participants 6, 11, 29 and 31 did not reach a study end-point.

c For participants 6 and 11, mutation A360V occurred in <25% of the viral population which is difficult to identify by population sequencing.

### Phenotypic analyses of HIV-1 containing A360V

To determine whether the A360V mutation conferred AZT resistance, we first cloned full-length RTs from participant samples 4, 11 and 29 into pxxLAI-3D, and selected a single clone for phenotypic analyses. Recombinant virus derived from participant sample 29 did not replicate in cell culture, and was excluded from subsequent analyses. The genotypes of the individual clones and population sequences obtained from the participant serum are shown in Table 2. The 360 V mutation in each of the two replication competent viruses was reverted to A360 by mutagenesis and the paired (A360/360 V) recombinants were tested for susceptibility to AZT (Table 4). Both recombinant viruses with RT sequences derived from on-treatment samples were resistant to AZT. Importantly, when the A360V mutation was reverted to wildtype, statistically significant decreases in AZT resistance were observed for each of the virus pairs (Table 4). We also generated a full-length RT and polymerase domain only bulk plasmid population for sample 3, since individual clones did not grow in cell-culture (Table 2, see Methods). Phenotypic analyses of these viruses further demonstrated that mutations in the connection (and/or RNase H) domain of this particular participant isolate contributed to AZT resistance. To further confirm the role of A360V in AZT resistance, we generated 5 site-directed mutant viruses in pxxLAI that contained A360V in 2 different backgrounds of TAMs (TAM-1 and D67N/K70R). AZT susceptibility assays (Table 5) revealed that the 360 V mutation alone confers 1.8-fold AZT resistance compared to wildtype (p<0.05). When A360V was in the context of TAM-1 or D67N/K70R, AZT resistance increased 1.9- and 2.1- fold, respectively (p<0.05).

**Table 4:** AZT susceptibility of recombinant viruses containing participant-derived RT sequences.

Sample ID	EC_50_ (µM)^a^	Fold-R^b^ (Mutant vs WT)	p-value^c^	Fold-R^d^ (360 V vs 360 A)	p-value^e^
Wildtype^f^	0.21±0.14	-	-	-	-
11Clone Revertant	0.83±0.18	4.0	0.0002	2.1	0.019
	0.39±0.11	1.9	0.0060		
4 Clone Revertant	9.09±2.30	43	<0.0001	1.6	0.048
	5.65±2.18	27	<0.0001		
3 Full-Length RT 3 Pol Domain RT	3.85±0.44	18	<0.0001	2.3	0.010
	1.67±0.46	7.9	<0.0001		

a Mean ± standard deviation from 3–11 independent experiments.

b Fold-resistance calculated by dividing EC_50_ of mutant virus by EC_50_ of wildtype (WT).

c Calculated using means of log_10_ transformed EC_50_ values and two-sided Student's *t* test.

d Fold-resistance calculated by dividing EC_50_ of 360 V virus by EC_50_ of 360 A virus.

e Calculated using means of log_10_ transformed EC_50_ values and two-sided Student's *t* test.

f Wildtype is xxLAI 3D (see Methods).

**Table 5:** AZT susceptibility of site-directed mutant HIV-1.

Site-Directed Mutant	EC_50_ (µM)^b^	Fold-R^c^ (WT vs Mutant)	p-value^d^	Fold-R^e^ (360 V vs 360 A)	p-value^f^
Wildtype (WT)^a^	0.23±0.11	-	-	-	-
A360V	0.41±0.11	1.8	0.022	-	-
D67N/K70R	1.22±0.50	5.3	<0.0001	1.9	0.022
D67N/K70R/A360V	2.30±0.88	10	<0.0001		
TAM-1	3.41±0.55	15	<0.0001	2.1	0.006
TAM-1/A360V	7.31±2.71	32	<0.0001		

a Wildtype (WT) is xxHIV-1_LAI_.

b Mean ± standard deviation from 4–6 independent experiments.

c Average fold-resistance (Fold-R) of site-directed mutant EC_50_ versus wildtype (WT).

d Calculated using means of log_10_ transformed EC_50_ values and two-sided Student's *t* test.

e Average Fold-R of 360 V versus A360 recombinant virus EC_50_.

f Calculated using means of log_10_ transformed EC_50_ values and two-sided Student's *t* test.

### Mechanism by which A360V confers AZT resistance

**Figure 1:**
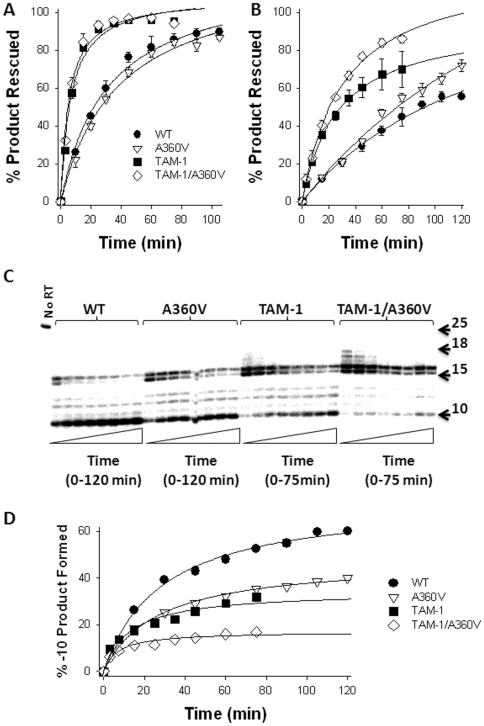
ATP-mediated AZT-MP excision activity and RNase H activity of wildtype, A360V, TAM-1 and TAM-1/A360V HIV-1 RT. **A**) Isotherms of ATP-mediated AZT-MP excision reactions carried out by wildtype and mutant HIV-1 RT on a DNA/DNA T/P. Data are the mean ± standard deviation from at least three independent experiments. Reaction times were: wildtype and A360V = 10, 20, 30, 45, 60, 75, 90, 105 min; TAM-1 and TAM-1/A360V = 3, 7.5, 15, 25, 35, 45, 60, 75 min. **B**) Isotherms of ATP-mediated AZT-MP excision reactions carried out by wildtype and mutant HIV-1 RT on an RNA/DNA T/P. Data are the mean ± standard deviation from at least three independent experiments. Reaction times were: wildtype and A360V = 15, 30, 45, 60, 75, 90, 105, 120 min; TAM-1 and TAM-1/A360V = 3, 7.5, 15, 25, 35, 45, 60, 75 min. **C**) Representative autoradiogram of the RNase H cleavage activity of the wildtype and mutant HIV-1 RTs. Experiments were carried out as described in the *Materials and Methods*. The reaction times were wildtype and A360V = 15, 30, 45, 60, 75, 90, 105, 120 min; TAM-1 and TAM-1/A360V = 3, 7.5, 15, 25, 35, 45, 60, 75 min. **D**) Isotherms for the accumulation of the −10 product formed by wildtype and mutant HIV-1 RT during AZT-MP excision.

TAMs in HIV-1 RT confer AZT resistance by enabling the enzyme to excise the chain-terminating AZT-MP moiety from the 3′-end of the DNA primer using ATP as a phosphate donor [7]. Previous biochemical studies demonstrated that the N348I and Q509L mutations in HIV-1 RT indirectly increase AZT resistance by decreasing the frequency of secondary RNase H cleavages that reduce the RNA/DNA duplex length of the T/P and diminish the efficiency of AZT-MP excision [13], [14], [23], [24], [26]. A similar phenotype has been proposed for the A360V mutation; however, the biochemical analyses reported used RTs that also contained N348I [26]. Therefore, we performed biochemical experiments to probe the mechanism(s) by which A360V decreased AZT susceptibility. In this regard, we first assessed the AZT-MP excision activity of the wildtype, TAM-1 and TAM4-1/A360V enzymes on a well-defined DNA/DNA and RNA/DNA T/P substrate that is routinely used in our laboratory [13], [14], [24], [26]. As described previously [13], [14], TAM-1 HIV-1 RT unblocked AZT-MP chain-terminated primers efficiently on both DNA/DNA and RNA/DNA T/Ps (Figure 1A, 1B). Introduction of the A360V mutation into either wildtype or TAM-1 RT increased the enzyme's excision activity on the RNA/DNA T/P but not on the DNA/DNA T/P (Figure 1A, 1B). We previously delineated the relationship between AZT-MP excision efficiency and RNase H activity on the RNA/DNA T/P substrate used in these experiments [23], [24]. These studies showed that the primary polymerase-dependent RNase H cleavage of RT does not impact the enzyme's AZT-MP excision efficiency, but polymerase-independent RNase H cleavages that reduce the RNA/DNA duplex length to less than 12 nucleotides abolish AZT-MP excision activity [23], [24]. In light of these data, we next evaluated the RNase H activity of the wildtype and mutant RTs that occurred during the ATP-mediated excision reactions described in Figure 1B. The A360V mutation was found to significantly reduce the frequency of a polymerase-independent cleavage event that decreases the RNA/DNA duplex to 10 nucleotides (Figure 1C, 1D). Taken together, these findings are consistent with the concept that A360V in HIV-1 RT impacts the efficiency of the AZT-MP excision reaction by an RNase H-dependent mechanism.

## Discussion

Recent studies show that RT inhibitors select for drug resistance mutations in the connection domain of RT including N348I and the A360V mutation reported here. The drug or drugs responsible for the selection of these connection domain mutations, however, are not well defined. For example, Yap *et al* reported that failure of AZT and nevirapine therapy was associated with the emergence of N348I, TAMs, and the non-nucleoside RT inhibitor resistance mutations K103N, V108I, Y181C/I and G190A/S [13]. By contrast, von Wyl *et al* reported that N348I predominantly emerged in participants receiving lamivudine and AZT [15], whereas Hachiya *et al* reported that N348I emerged on AZT- and/or ddI-containing therapies [27]. Of note, the A360V mutation has not been associated with failure of any specific drug combinations [28]. The limitation of these prior studies is that they relied on retrospective analyses of sequences in clinical databases. As such, RT sequences were obtained from participants exposed to multiple RT inhibitors, without pretherapy or longitudinal sequences available, making it difficult to pinpoint the specific drug or drugs responsible for mutation selection.

In the current study, we compared paired pre- and on-therapy full-length RT sequences from 23 participants who had received only AZT monotherapy in ACTG study 175. Our analyses reveal that the A360V mutation in the connection domain emerged with similar or higher frequency as TAMs M41L, D67N, L210W and K219E/Q. Longitudinal analyses also showed that A360V was detected at the same time or after the appearance of TAMs but not before. Importantly, we also show that the A360V mutation confers ∼2.0-fold AZT resistance both in the full-length RT sequence context it was selected *in vivo* and in the xxLAI molecular clone. This is the first study to take a systematic experimental approach to determine whether AZT alone selects for mutations in the C-terminal domains of HIV-1 RT. As a result, both the selection of A360V during AZT monotherapy and resistance to AZT from A360V are unequivocally shown.

We previously reported that in *in vitro* selection experiments HIV-1 selected for two novel mutations – A371V in the connection domain and Q509L in the RNase H domain – in combination with D67N, K70R and T215F that together conferred greater than 100-fold AZT resistance [16]. The A360V mutation was not selected. In the 23 on-treatment samples sequenced in the current study, we noted a strong trend (p = 0.06) toward selection of A371V with AZT monotherapy. By contrast, the Q509L mutation was not present in any of the sequences. These findings indicate that the drug pressure and other environmental factors that promote resistance selection differ between participants and cell culture and the results of *in vitro* experiments cannot always be extrapolated *in vivo*.

Of note, the N348I mutation was not identified in any of the sequences in this study. Although our sample size is small (N = 23), this is surprising given that N348I and A360V mutations decrease AZT susceptibility to the same extent (this study, [13]). Furthermore, the phenotypic mechanisms by which these mutations confer AZT resistance are similar (this study, [13], [26]) in that they both diminish the frequency of secondary RNase H cleavage events that reduce the RNA/DNA duplex length of the T/P and diminish the efficiency of AZT-MP excision. Additional virological and biochemical analyses may provide insights into this conundrum.

In summary, this study of full-length RT sequences from paired pre- and post-therapy samples shows that the A360V mutation in the connection domain of RT emerges on AZT monotherapy with similar frequency as TAMs in the polymerase domain and confers ∼2-fold resistance to AZT through an excision-promoting molecular mechanism. These findings provide additional insight into the importance of the connection domain of RT in resistance to NRTI and NNRTI.

## Materials and Methods

### Cells and Reagents

MT-2 cells were cultured in RPMI 1640 (Whittaker MA Bioproducts, Walkersville, MD) supplemented with 10% fetal bovine serum (FBS), 2 mM L-glutamine, 10 mM HEPES buffer, 50 IU/mL of penicillin and 50 µg/mL of streptomycin. The P4/R5 HeLa reporter cell line was maintained in Dulbecco's Modified Eagle Medium, Phenol Red Free (Gibco-BRL, Grand Island, NY) supplemented with 10% FBS, 50 IU/mL of penicillin, 50 µg/mL of streptomycin and 0.5 µg/mL of puromycin (Clontech, Palo Alto, CA). P4/R5 cells were provided by Dr. Nathaniel Landau (NYU School of Medicine, New York, NY). MT-2 cells and AZT were obtained from the NIH AIDS Research and Reference Reagent Program. AZT-TP was purchased from TriLink Biotechnologies (San Diego, CA). RNA and DNA oligonucleotides were synthesized by Integrated DNA Technologies (Coralville, IA).

### Participant Samples

All samples were obtained from participants enrolled in the AIDS Clinical Trials Group (ACTG) study 175 (ClinicalTrials.gov number, NCT00000625) [17]. ACTG 175 was a randomized, double-blind, placebo-controlled trial designed to compare AZT or didanosine monotherapy to AZT/didanosine or AZT/zalcitabine combination therapy. Participants provided written informed consent and the study was approved at each site by an institutional review board. In Text S1, we list the institutions and investigators that participated in ACTG 175. ACTG approval was sought for the current study and was obtained through the appropriate New Works Concept Sheet (NWCS 304).The primary study endpoint was defined by: i) confirmed CD4 count 50% below the average of two pre-treatment counts; ii) AIDS related endpoints defined by the 1987 CDC criteria [18]; or iii) death.

For the current study, samples were selected from participants who were randomized to receive AZT monotherapy, had reached a study endpoint and for whom stored serum samples were available at pre-therapy (week 0) and from at least 1 of 3 longitudinal time points (weeks 8, 20 or 32). Samples were also obtained from participants who were randomized to receive AZT monotherapy but did not reach a study endpoint. HIV-1 RNA in serum was quantified using the Roche Amplicor Monitor HIV-1 Ultrasensitive Assay Kit 1.5 (Roche Diagnostics Corporation, Indianapolis, IN).

### Full-length RT sequencing

Viral RNA was extracted from serum samples using the QIAamp® Viral RNA Mini kit (Qiagen, Valencia, CA). RNA was converted to cDNA and full-length HIV-1 RT was amplified using SuperScript™ III One-Step RT-PCR System with Platinum® *Taq* High Fidelity (Invitrogen, Carlsbad, CA) using first-round RT-PCR primers: 5′-AGGAAGAT GGAAACCAAAAATGATAG-3′ and 5′-CCTTGACTTTGGG GATTGTAGGGAA-3′ and second round PCR primers: 5′-AGGAAGATGGAAACCAA A AATGATAG-3′ and 5′-CACAGCTGGCTACTATTTCTTTGC- 3′. PCR products were purified with ExoSAP-IT® (USB, Cleveland, OH), population sequenced using the Big Dye terminator kit (v.3.1) on an ABI 3100 automated DNA sequencer (Applied Biosystems, Foster City, CA) and compared to consensus subtype B RT (Los Alamos HIV Sequence Database; http://www.hiv.lanl.gov). Bidirectional sequences were assembled and analyzed using Sequencher 4.9 software (Gene Codes Corporation, Ann Arbor, MI). Sequencing peaks that were greater than 25% of the total peak heights were counted as mutations. All sequences were HIV-1 subtype B (REGA HIV-1 Subtyping Tool Version 2.0 [19]).

### Production of infectious recombinant virus containing participant-derived RT sequences

The full-length infectious HIV-1 clone pxxLAI [20] was modified by site-directed mutagenesis to introduce silent mutations into RT at codons 321–323, 358, 417–418, 554 and in integrase at codons 29–31 to generate the unique restriction sites Bst*BI*, Mlu*I*, Hpa*I*, Ngo*MIV* and Sgr*AI*, respectively (pxxLAI-3D). Next, an RT defective cloning vector pxxLAI-3DΔnp was developed by deletion of RT polymerase domain codons 113 to 315, using restriction sites Nsi*I* to Pflm*I*, as described previously [20]. To clone participant derived full-length RTs into pxxLAI-3DΔnp, the restriction enzyme sites Bcl*I* and Sgr*AI* were first added to the 5′ and 3′ ends of genotyping amplicons using the primers: Bcl-forward (5′-GTTTTATCAAAGTAAGACAGTATGATCAGATAC-3′) and Sgr-reverse (5′- CTACCACCGGTGGCAGGTTA -3′). PCR products and pxxLAI-3DΔnp were digested with *Bcl*I and *Sgr*AI to form 1.89 kb full-length RT inserts and a 9.87 kb pxxLAI-3D cloning vector. Vector and full-length RT inserts were ligated with T4 DNA ligase (NEB, Ipswich, MA) and transformed into C2925 cells (NEB, Ipswich, MA). Individual recombinant clones or bulk plasmid populations were isolated and DNA sequenced to confirm similarity with the RT sequences in the serum sample from which they were derived. To clone the DNA polymerase domain of participant derived RTs into pxxLAI-3DΔnp, the restriction enzyme sites Bcl*I* and Bst*BI* were first added to the 5′ and 3′ ends of genotyping amplicons using the primers: Bcl-forward (5′-GTTTTATCAAAGTAAGACAGTATGATCAGATAC-3′) and Bst-reverse (5′- CTATTAAGTCTTTCGAAGGGTCATAATAC -3′). PCR products and pxxLAI-3DΔnp were digested with Bcl*I* and Bst*BI* to form 1.08 kb full-length RT inserts and a 10.68 kb pxxLAI-3D cloning vector. Vector and full-length RT inserts were ligated and transformed, as described above. The bulk plasmid population of participant-derived, RT polymerase domain recombinant clones was isolated and DNA sequenced to confirm similarity with the RT sequences in the serum sample from which they were derived.

Site-directed mutagenesis (QuickChange® II XL Site-Directed Mutagenesis Kit; Agilent, La Jolla, CA) was used to revert the 360 V mutation (codon GTC) to the wildtype amino acid (360 A; codon GCC) in participant-derived full-length recombinant clones. Individual recombinant clones were then isolated and sequenced to confirm reversion. Infectious virus for AZT susceptibility assays was generated and titered as described previously [21].

### Production of site-directed mutants containing A360V

The A360V mutation was also introduced by site-directed mutagenesis (QuickChange® II Site-Directed Mutagenesis Kit; Agilent, La Jolla, CA) into the backbone of wildtype HIV-1 RT and into RTs that contained the *D67N/K70R or* M41L/L210W/T215Y (TAM-1) mutations in the p6HRT plasmid. The mutated RT sequences were then cloned into pxxLAI and infectious virus was generated and titered as described previously [21].

### Drug susceptibility testing

The AZT susceptibility of participant-derived recombinant virus and site-directed mutants were determined in P4/R5 cells as described previously [21]. Briefly, three-fold dilutions of inhibitor were added to P4/R5 cells in triplicate and cells were infected with an amount of virus that produced ∼100 relative units of light (RLU) in no drug control wells. After 48 hours, cells were lysed (Gal-Screen; Tropix/Applied Biosystems, Foster City, CA) and the RLU was measured using a ThermoLabSystems luminometer (Waltham, MA). The concentration of drug required to inhibit viral replication by 50% (EC_50_) was determined from 3–11 independent experiments.

### Biochemical analyses of recombinant HIV-1 RT containing A360V

HIV-1 RTs containing the A360V, TAM-1 or TAM-1/A360V mutations were purified to homogeneity as described previously [22]. The ability of the recombinant enzymes to facilitate the ATP-mediated removal of AZT-monophosphate (MP) from chain-terminated DNA/DNA and RNA/DNA template/primers (T/Ps) using assay systems and conditions that have been studied extensively in the Sluis-Cremer laboratory [13], [14], [23], [24]. For these assays a 26-nucleotide DNA primer (pr26; 5′-CCTGTTCGGGCGCCACT GCTAGAGAT-3′) was 5′-radiolabeled with [γ-^32^P]ATP and chain-terminated with AZT-MP to generate P_AZT_. P_AZT_ was then annealed to either a 35-nucleotide DNA (T_DNA_; 5′-AGAATGGAAAATCTCTAGCAGT GGCGCfoCCGAACAG-3′) or RNA (T_RNA_: 5′-AGAAUGGAAAAUCUCUAGCAGUGGCGCCCGAACAG-3′) template. Excision assays were carried out by first incubating 20 nM T_RNA_/P_AZT_ or T_DNA_/P_AZT_ with 3 mM ATP, 10 mM MgCl_2_, 1 µM dTTP and 10 µM ddCTP in a buffer containing 50 mM Tris-HCl (pH 7.5) and 50 mM KCl. Reactions were initiated by the addition of 200 nM wildtype or mutant RT. Aliquots were removed at defined times and analyzed as described previously [13], [14], [23], [24]. RNase H activity was evaluated using the same AZT-MP chain-terminated RNA/DNA T/P substrate described above, except the 5′-end of the RNA was ^32^P-end-labelled. Assays were carried out using 20 nM T_RNA_/P_AZT_, 3 mM ATP and 10 mM MgCl_2_ in a buffer containing 50 mM Tris-HCl (pH 7.5) and 50 mM KCl. Reactions were initiated by the addition of 200 nM wildtype or mutant HIV-1 RT.

### Statistical Analyses

The statistical significance of HIV-1 RNA levels in pre-therapy and last-on-therapy serum samples was calculated using the Mann-Whitney rank sum test. To identify mutations selected by AZT monotherapy, paired pre-therapy and last-on-therapy sequences from each participant were examined for known NRTI resistance mutations in the polymerase domain using the International AIDS Society – USA (IAS-USA) 2010 resistance table [25] and for novel mutations in the polymerase, connection and the RNase H domains of RT. The statistical significance (p<0.05) of differences in mutant frequency between pre-therapy and last-on-therapy sequences was determined using a two-sided exact McNemar's test. Due to small sample size, statistical significance was determined using N = 23 (5 participants who reached a study endpoint and 18 participants who did not) and p-values were unadjusted for multiple comparisons. For drug susceptibility data, the concentration of drug required to inhibit viral replication by 50% (EC_50_) were log_10_ transformed and compared for significant differences (p-value<0.05) using the two-sample Student's *t* test.

## Supporting Information

Text S1 **AIDS Clinical Trials Group Study 175 Protocol Team.**
(DOCX)
