# Supplementary material for: Zidovudine (AZT) Monotherapy Selects for the A360V Mutation in the Connection Domain of HIV-1 Reverse Transcriptase
Source: PLoS One. 2012 Feb 21;7(2):e31558. doi: 10.1371/journal.pone.0031558 (PMC3283647; doi:10.1371/journal.pone.0031558)
Supplement: Text S1 — AIDS Clinical Trials Group Study 175 Protocol Team. (DOCX) [file pone.0031558.s001.docx]

## Text S1: AIDS Clinical Trials Group Study 175 Protocol Team

The following institutions and investigators participated in ACTG 175: **Harvard University** — C. Crumpacker, D. Craven, B. Chapman, C. Grodman; **Case Western Reserve University** — M. Chance, K. Citraro, A. Davidson; **Northwestern University**— R. Hirschtick, J. Pottage, Jr., J. Pulvirenti; **University of Minnesota** — H. Balfour, N. Reed, S. Swindells, R. Nelson; **Mt. Sinai Medical Center** — D. Mildvan, J. Hassett, B. Simpson, K. Luyks; **University of California, San Diego** — D. Richman, S. Spector, C. Jacobsen; **Stanford University** — V. Tallman, M. Rinki, D. Carroll, G. Van Raalte; **University of California, Los Angeles** — R. Mitsuyasu, G. Beall, W. Hardy, G. Mathisen; **University of Washington, Seattle** — A. Collier, B. Royer, M. Paradise, L. Sacks; **Washington University** — W. Powderly, A. Slack, T. Stiffler, M. Royal; **Ohio State University** — M. Para, N. Stark, C. Jackson, J. Neidig; **University of North Carolina** — J. Eron, T. Lane, J. Horton, D. Ragan; **University of California, San Francisco** — D. Abrams, L. Johnson, K. Dybek, J. Carroll; **University of Rochester** — R. Reichman, C. Greisberger, R. Hewitt, D. Blair; **Indiana University** — K. Fife, M. Goldman, K. Todd, B. Zwickl; **University of Colorado** — D. Kuritzkes, V. Waite, M. Ray; **Charity Hospital** — N. Hyslop, Jr., D. Mushatt, R. Clark, J. Zachary; **University of Cincinnati** — B. Wong, J. Brinkdopke, B. Jackson, D. Dayton; **Albert Einstein Medical Center** — R. Soeiro, D. Stein, J. Schliosberg, B. Zingman; **State University of New York–Stonybrook** — R. Steigbigel, J. Fuhrer, P. Mariuz, C. Wallace; **University of Southern California** — J. Geiseler, J. Leedom, S. Cordina, C. Olson; **University of Miami** — M. Fischl, D. Jayaweera, J. Patrone Reese, E. Dale; **Cornell University Medical Center** — K. Sepkowitz, V. Sharp, D. Shepp; **Johns Hopkins University** — J. Bartlett, R. Becker, D. Baker, D. Wright; **Hershey Medical Center** — W. Ehmann, J. Zurlo, M. Kreher, F. Damianos; **University of Massachusetts** — S. Cheeseman, J. Avato, C. Bova, M. Sands; **St. Luke’s–Roosevelt Hospital Center** and **Columbia University** — M. Grieco, G. McKinley, J. Rivera, J. O’Connor; **University of Alabama, Birmingham** — D. Davis, K. Squires, J. Gnann, M. Saag; **University of Texas Medical Branch, Galveston** — R. Pollard, M. Borucki, K. Waterman, G. Casey; **University of Pennsylvania, Philadelphia** — I. Frank, D. Dunbar, I. Matozzo, S. Hauptman; **New York University Medical Center** — V. McAuliffe, V. Rosenwald, F. Valentine; **University of Medicine and Dentistry of New Jersey–University Hospital** — P. Kloser, P. Correll; **Duke University Medical Center** — J. Bartlett, R. Dodge, P. Robinson, K. Shipp; **Yale University** — G. Friedland, E. Cooney, M. Fiellin, B. Griffith; **University of Puerto Rico** — G. Vazquez, M. Cruz-Ortiz, V. Ramirez, I. Lopez; **Howard University** — W. Greaves, J. McNeil, R. Delapenha, V. Holley-Trimmer; **University of Hawaii** — M. Heath- Chiozzi, D. Ogata-Arakaki, S. Bon Akina, M. Millard; **Georgetown University** — P. Pierce, P. Kumar, J. Timpone, L. Green; **Boston Children’s Hospital** — K. McIntosh, A. Rubin-Hale, K. Knox-Burke, H. Mahoney-West; **Montefiore Adolescent AIDS Program** — D. Futterman, D. Monte, N. Hoffman; **State University of New York–Brooklyn** — K. Chirgwin, D. Smith, G. Garner, D. Hauck; **Tulane University** — R. Van Dyke, S. Abdalian, C. Degenstein, J. Price; **National Hemophilia Foundation** — D. Brettler, F. Rickles, P. Smith, C. Cornell, S. Seremetis, R. Lipton, L. Cisar, S. Arkin, M. Ragni, J. Steinberg, C. Lessinger, W. Hanna, T. Hadley, J. Penner, S. Adair, E. Czapek, H. Ritter, W. Hoots, M. Cantini, C. Rutherford, D. MacFarlane, R. Stuart, S. Stabler, S. Giambartolomei, M. Ford, G. Gjerset, J. Goldsmith, A. Sosa, N. Sanders; **ACTG Operations Office** — P. Kasdan, J. Cook, B. Landry, L. Nerhood; **Data Management Center** — M. Colon-Ruiz, K. Ojudun; **Division of AIDS, National Institutes of Health** — A. Martinez, C. Pettinelli.
